# Carbon benefits from protected areas in the conterminous United States

**DOI:** 10.1186/1750-0680-8-4

**Published:** 2013-04-17

**Authors:** Daolan Zheng, Linda S Heath, Mark J Ducey

**Affiliations:** 1 Department of Natural Resources & the Environment, University of New Hampshire, Durham, New Hampshire, 03824, USA; 2 USDA Forest Service, Northern Research Station, Durham, New Hampshire, 03824, USA

**Keywords:** Afforestation and deforestation, Net deforestation rate, Protected and unprotected forestlands, Forest carbon emissions, Forest carbon sequestration

## Abstract

**Background:**

Conversion of forests to other land cover or land use releases the carbon stored in the forests and reduces carbon sequestration potential of the land. The rate of forest conversion could be reduced by establishing protected areas for biological diversity and other conservation goals. The purpose of this study is to quantify the efficiency and potential of forest land protection for mitigating GHG emissions.

**Results:**

The analysis of related national-level datasets shows that during the period of 1992–2001 net forest losses in protected areas were small as compared to those in unprotected areas: -0.74% and −4.07%, respectively. If forest loss rates in protected and unprotected area had been similar, then forest losses in the protected forestlands would be larger by 870 km^2^/yr forests, that corresponds to release of 7 Tg C/yr (1 Tg=10^12^ g). Conversely, and continuing to assume no leakage effects or interactions of prices and harvest levels, about 1,200 km^2^/yr forests could have remained forest during the period of 1992–2001 if net area loss rate in the forestland outside protected areas was reduced by 20%. Not counting carbon in harvested wood products, this is equivalent to reducing fossil-fuel based carbon emissions by 10 Tg C/yr during this period. The South and West had much higher potentials to mitigate GHG emission from reducing loss rates in unprotected forests than that of North region. Spatially, rates of forest loss were higher across the coastal states in the southeastern US than would be expected from their population change, while interior states in the northern US experienced less forest area loss than would have been expected given their demographic characteristics.

**Conclusions:**

The estimated carbon benefit from the reduced forest loss based on current protected areas is 7 Tg C/yr, equivalent to the average carbon benefit per year for a previously proposed ten-year $110 million per year tree planting program scenario in the US. If there had been a program that could have reduced forest area loss by 20% in unprotected forestlands during 1992–2001, collectively the benefits from reduced forest loss would be equal to 9.4% of current net forest ecosystem carbon sequestration in the conterminous US.

## Background

Forest loss is a significant contributor to accumulation of greenhouse gases (GHGs) in the atmosphere by releasing carbon stored in vegetation and soils. Conversion of forests to other land use or land cover not only releases the carbon stored in the forests, but also reduces forest carbon sequestration. In the tropics, primary forestlands designated as protected areas which exclude human occupation or exploitation for resources and conserve biological diversity have been shown to have lower rates of forest loss than those without protection status [1-3]. Thus, establishing protected areas for biodiversity in primary forests can also provide multiple benefits including climate change mitigation, reduction in forest degradation, and reduction in forest area change [1-4]. Other approaches, such as directly reducing the drivers of deforestation or by strengthening land tenure systems, can also reduce forest conversion.

For managed temperate forests such as in the United States, other major carbon management options to reduce carbon emissions include: 1) keeping forests as forests (i.e., reducing forest conversion to nonforest), 2) reforesting areas where forests historically occurred, 3) burning forest biomass as a substitute for fossil fuel, and 4) upholding forest-derived products such as wood-framed buildings [5]. These options are seen as a practical and low-cost strategy to climate mitigation [6-8]. However, studies that quantify forest area change effects in temperate forests associated with protection status for biodiversity benefits over large scales and that also estimate associated carbon emissions or benefits are lacking. Estimates of carbon benefits resulting from protection status would be useful to inform management or policy-level decisions.

Although some project-level methodologies are available for estimating carbon and biodiversity benefits [9], development of approaches for national-level estimates are still in the early stages. Remote sensing observations have been used as the basis for providing a useful, reliable, and consistent tool for monitoring land-cover changes and forest area dynamics [10-14]. DeFries et al. [15] concluded that analysis of remotely sensed data is the only practical approach to measure changes in forest area at national and international scales because changes in forest area since the early 1990s can be observed from space with consistency and confidence. However, remotely sensed data have both advantages and limitations compared to ground-based inventory data, such as data from the USDA Forest Inventory and Analysis (FIA) program, and the results and their interpretation for policy can differ [16]. For example, the definition of forestland in satellite-derived National Land-Cover Dataset (NLCD) is based on land cover whereas forest area determined in the FIA dataset is based on forestland use [17].

We used remote sensing derived land cover data to estimate changes in forest area in this study because these data were available consistently and contiguously over the conterminous US over the time period of study. Results may not be strictly compatible with estimates of forest area change derived from land use statistics. In the other words, land use is a more complicated concept that also involves social and economic perspectives within which lands are managed [18]. However, high correlations (r > 0.82) were observed between NLCD- and FIA-based forest area estimates at the state level in both years of 1992 and 2001, respectively [17].

Protection is thought to be most immediately beneficial in those areas which are under the greatest risk of being directly impacted by human population, although population is not the only factor affecting land cover change. Some studies have demonstrated generally negative relationships between population growth and forest cover change worldwide during the last two-three decades, although regional variations exist [19-21]. We hypothesize that: 1) forest area change in the US differs according to protection status; and 2) effects of land protection status vary depending on the population attributes of the surrounding area.

The overall goal of this study is to estimate the carbon benefits due to protected area designation for forests of the conterminous US by examining forest loss rates in different protection status as well as potential reduction in loss rates within unprotected forestlands. Four specific objectives are to: 1) illustrate and compare differences in area change (from 1992 to 2001 as baseline information) between protected and unprotected forestlands and estimate corresponding carbon benefits; 2) estimate the effects that the reduction in forest area loss in protected forestlands has on mitigation of carbon emissions; 3) examine, as a hypothetical scenario, how much forest area loss might be reduced and its potential to benefit carbon mitigation; and 4) reveal how demographic characteristics are generally related to forest area dynamics in protected and unprotected forestlands.

## Results

### Forest area change and protection status

**Figure 1:**
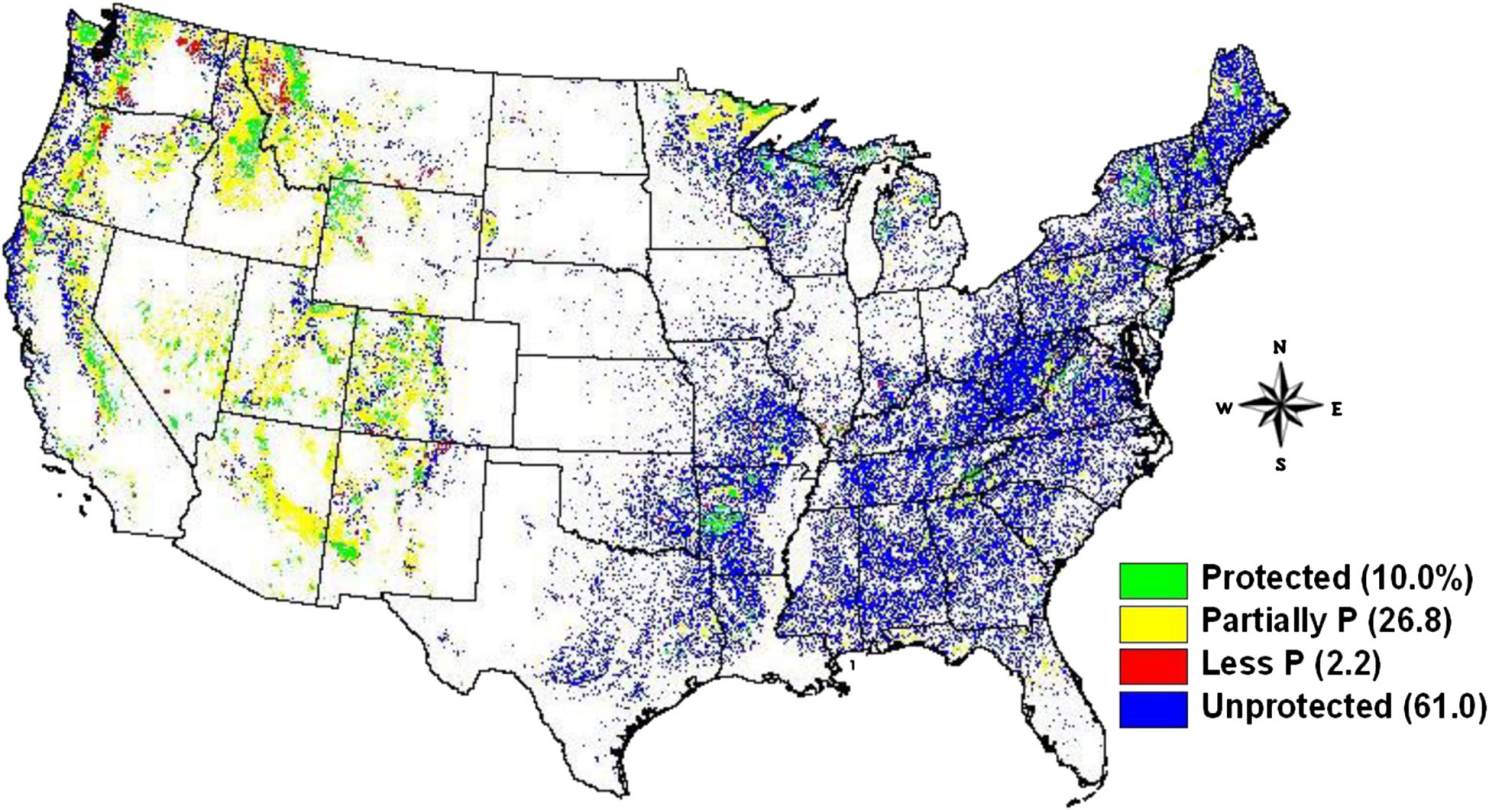
**Spatial distribution of generalized land protection status involving forestlands in the conterminous US.** Protected = Gap codes 1 & 2, Partial Protected = Gap code 3, and Less Protected = Gap code 4 of the PADUS 1.1 [36]. Numbers in the parentheses are frequency distribution. Unprotected forestlands were obtained after subtracting all forestlands contained in the PADUS 1.1 from all US conterminous forestlands in 2001 obtained from the change map [14].

Approximately 10% (or 204,000 km^2^) of the forestland in the 48 states of the US was in protected areas (under GAP codes 1 and 2, Figure 1) in 2001, with 1.3 million km^2^ of unprotected forests. These protected area forests were unevenly distributed in the conterminous US. About 59% of protected forests occurred in the western 11 states, accounting for 17% of total forestland in those states. Among the remaining 37 states, protected forests only accounted for 6.2% of total forestland (Figure 1).

The average net forest area loss between 1992 and 2001 in the conterminous US was about 0.3% per year in relation to the total forestland based on the Retrofit Change Map [14,17]. Overall, we found that the area-weighted net forest area change rate in the conterminous US’s unprotected forests was about −0.45% per year, compared to the −0.08% per year in the protected forests for the 9-year study period (Table 1). These rates differed significantly (Friedman test chi-squared=55.29, df=2, p<0.0001). Pairwise comparisons showed highly significant differences (p<0.0001) between protected and unprotected lands, as well as between partially protected and unprotected lands. However, the difference between fully and partially protected lands was not statistically significant (p=0.91). Expressed on an annual basis, the mean forest area change rate for protected forests was −0.08% per year, or 82% lower than that in the unprotected forestland (−0.45% per year).

**Table 1:** **Estimated net area change rates in percent (calculated as (Area**_**aff **_**– Area**_**def**_**) / Area**_**1992 **_**x 100) in protected (Pro) and unprotected (Unp) forests by state in the conterminous U.S based on the NLCD Retrofit Change Map (1992–2001), and the PADUS 1.1 dataset; and forest areas (km**^**2**^**) as described**

			**Forest Area (km**^**2**^**)**		
**State**	**Pro. Rate %**	**Unp. Rate %**	**In Protected Area**^**1**^	**Due to Protection**^**2**^	**In Unprotected Scenario**^**3**^
Alabama^+^	−4.04	−6.33	1,045	25	906
Arizona*	−1.53	−7.56	6,632	406	40
Arkansas^+^	−0.06	−4.67	8,411	388	498
California*	−1.31	−2.42	17,826	202	187
Colorado*	−1.97	−8.22	14,009	894	324
Connecticut	−0.64	−2.47	310	6	31
Delaware^#^	−6.9	−5.34	54	NA	12
Florida^+^	−6.13	−7.85	704	13	388
Georgia^+^	−3.66	−7.95	2,238	100	1,144
Idaho*	−3.4	−4.69	11,037	147	110
Illinois	1	−1.83	2,021	57	68
Indiana	0.54	−0.56	1,857	20	20
Iowa	−1.77	−1.78	278	0	32
Kansas^$^	3.41	1.5	91	NA	NA
Kentucky^+^	−0.75	−3.19	1,316	32	325
Louisiana^+^	−0.94	−8.69	1,586	124	438
Maine	−1.29	−3.08	1,917	35	304
Maryland	−1.5	−2.92	655	9	45
Massachusetts	−0.37	−3.87	540	19	63
Michigan^$^	1.43	0.67	10,034	NA	NA
Minnesota^$^	1.16	0.37	6,084	NA	NA
Mississippi^+^	−2.07	−5.06	378	12	477
Missouri	0.22	−2.47	3,589	97	290
Montana*	−0.38	−4.7	13,526	587	138
Nebraska^#^	−4.19	−1.23	183	NA	8
Nevada*	−1.03	−17.21	7,008	1,146	47
New Hampshire	0.1	−1.27	2,044	28	32
New Jersey	−1.04	−2.95	2,192	42	23
New Mexico*	−0.5	−1.26	8,780	67	27
New York	−0.22	−1.95	10,259	178	196
North Carolina^+^	2.09	−5.7	1,760	134	603
North Dakota^$^	1.22	0.63	332	NA	NA
Ohio	0.51	−1.95	587	14	121
Oklahoma^+^	0.65	−3.57	1,554	65	257
Oregon*	−2.09	−9.41	8,423	629	613
Pennsylvania	−0.1	−1.28	2,051	24	140
Rhode Island	−1.41	−4.69	70	2	9
South Carolina^+^	−3.39	−8.32	713	36	540
South Dakota^#^	−7.51	−2.9	480	NA	11
Tennessee^+^	−0.56	−4.4	3,019	117	448
Texas^+^	0.38	−6.87	1,326	96	1,002
Utah*	−1.09	−3.6	8,969	227	74
Vermont	0.1	−0.78	957	8	21
Virginia^+^	1.58	−3.3	3,413	177	347
Washington*	−0.26	−5.55	13,982	740	288
West Virginia	−0.4	−1.1	2,476	17	98
Wisconsin	−0.44	−0.92	7,088	35	88
Wyoming*	0.44	−8.43	9,897	874	88
Area change^4^	−0.74	−4.07			
Mean rate^5^ (%)	−1	−3.98			
Std.	2.17	3.4			
US48			203,701	7,829	10,921

^1^ Amount of 2001 forestland (km^2^) in protected areas.

^2^ Forest area (km^2^) that would be reduced in the protected forests if net forest area loss rates in the corresponding unprotected forests (i.e., within the same state) were applied.

^3^ How much forest area (km^2^) could be increased in the unprotected forests if net forest loss rates were reduced by 20%. Negative rate indicates a forest loss whereas positive rate suggests a forest gain.

^4^ Area weighted.

^5^ Mean forest area change rate of the 48 states and standard deviation were both in percent.

^+^ 13 Southern states (see Figure 2).

^*^ 11 Western states. The remaining states were in the Northern region.

^#^ States where forest area loss rates in the protected forests were larger than those in the unprotected forests. Therefore, calculation of presumed additional area loss in the protected forests using forest area loss rates in the corresponding unprotected forests (i.e., within the same state) were not applicable (NA).

^$^ States gained forest areas during the period, thus, no reduction in forest area loss is applicable (NA).

Over the 9-year period, approximately 7,800 km^2^ (≈ 870 km^2^ per year) more forestland would have been converted to nonforest in currently protected forests, if those forests had area dynamics similar to those of unprotected forests (Table 1). Conversely, the scenario analysis indicated that about 10,900 km^2^ forestland in unprotected areas would remain forestland during the period if the current forest area loss rate were reduced by 20% (Table 1).

**Figure 2:**
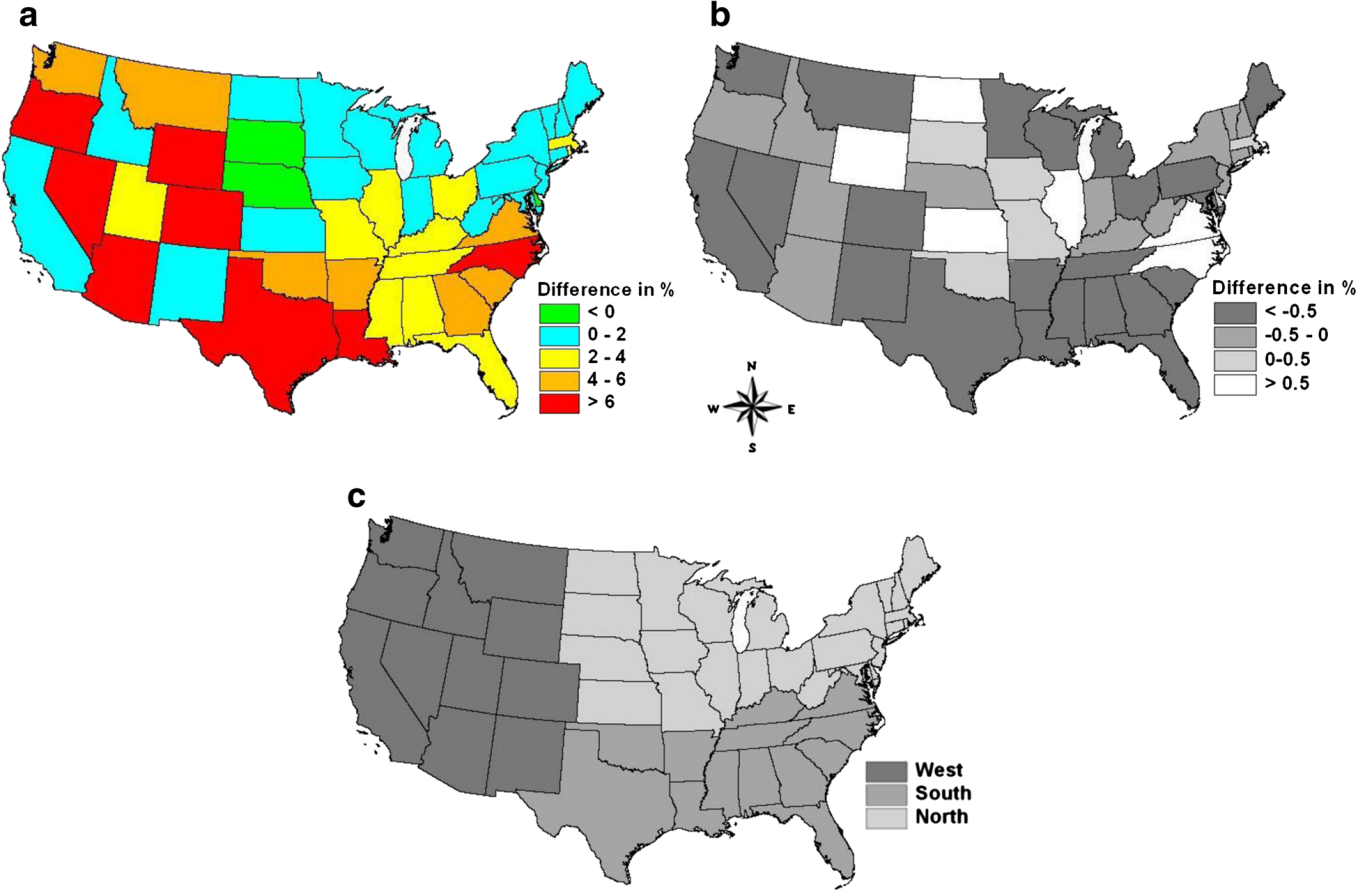
**a) Difference in net forest area loss rates between protected and unprotected forestlands:** calculated as Rate_protected_ - Rate_unprotected_ based on the numbers shown in Table 1 for each state. In general, the larger the difference the greater the reduction in forest area loss due to the adoption of protected areas; **b) Difference in afforestation rates between protected and unprotected forestlands:** calculated as AffRate_protected_ - AffRate_unprotected_ for each state (data not shown). Positive numbers indicate the afforestation rate in protected areas was greater than the corresponding rate in unprotected areas for a given state, and the negative numbers suggest the opposite; and **c) Regional division used for summary.**

In four states (Kansas, Michigan, Minnesota, and North Dakota), both protected and unprotected forestlands gained forest area during the period (Table 1), but the protected areas gained more forest than the unprotected areas. Only three states indicated that protected areas had a greater rate of forest loss than unprotected areas: Delaware, Nebraska, and South Dakota. In all other states, net loss rates in protected forests were lower than those in unprotected forests (Figure 2a). Eighty-eight percent of total gross forest area loss in the 48 states occurred in the unprotected forestland.

At the regional level, the Northern region had the lowest net forest area change rates per year in both protected and unprotected forestlands (−0.75% and −1.76%), and consequently, had the smallest difference in terms of forest area change rate between these two statuses (Table 2). The Southern region had the highest forest area change rate in protected forestland (−1.30%) and the Western region showed the highest change rate in unprotected forestland (−6.64%). Much higher variation in net forest area change rate was seen in unprotected forests than in protected forests in the Western region. However, the patterns of variation between protected and unprotected were reversed in the Northern and Southern regions (Table 2). A partial explanation for this may be the degree of ownership mixture within a given protection status, as discussed below.

**Table 2:** **Net forest area change rates (%), areas (km**^**2**^**), and carbon benefits (Tg C) for protected (Prot) and unprotected (Unprot) forestlands by the three geographic regions (defined in Figure**2c) during the 9-year period^*^

				**Unprotected**	**C**	**Protected**	**C Benefits**
	**Prot**	**Unprot**	**Difference**	**Forest Area**^**1**^	**Mitigation**	**Forest Area**	**From**
**Region**	**(%)**	**(%)**	**(%)**	**(km**^**2**^**)**	**(Tg)**	**(km**^**2**^**)**	**Prot. (Tg)**^**2**^
North	−0.75	−1.76	1.01	507,734	13.897	56,200	5.417
	(2.44)	(1.67)					
South	−1.30	−5.84	4.54	605,483	49.055	27,500	9.123
	(2.42)	(1.97)					
West	−1.19	−6.64	5.45	163,775	22.301	120,400	52.059
	(1.05)	(4.37)					

^*^ The change rates were **c**alculated as the average of state means within a given region. Difference was estimated as Rate_prot_ - Rate_unprot_. Thus, the more positive the difference, the greater the potential in climate change mitigation by adjusting current forest area loss rates in unprotected forests. The numbers in the parentheses are standard deviation, also in percentage. Carbon (C) mitigation (including both reduced emissions and additional fixation) and C benefits from protection are both in teragrams.

^1^ Area calculated from the PADUS1.1 layer and the NLCD Retrofit Change Map in 2001, in which applying 20% reduction in net forest becoming nonforest rate would result in corresponding amounts of carbon mitigation shown on the next column.

^2^ The carbon benefit attributed to the protected areas in each region is based on the reduced forest area loss in protected area forests and the associated carbon.

To examine consistency between patterns of afforestation and gross forest loss by protection status, we calculated both rates at the state level. Protection is not only associated with lower gross forest area loss rates, but also with lower afforestation rates in general. The majority of the states (36 out of the 48) had lower afforestation rates in the protected areas than those in the unprotected areas except a dozen of states focused in the middle of the conterminous US (Figure 2b). These states tend to have large areas of nonforest. Across the conterminous US, afforestation rates were 0.76% and 2.25% in protected and unprotected forestlands respectively, during the 9-year period. The corresponding gross forest area loss rates (without afforestation) were about −1.50% and −6.30% in protected and unprotected forestlands for the study period, respectively. These rates undoubtedly reflect the relative prevalence of different disturbance types, such as fire and harvest, during the 1990s. No clear relationships were observed between afforestation rate and population growth in either protection status.

### Potential effects of reducing forest area loss on carbon mitigation

Reducing forest area loss can affect forest carbon dynamics through two pathways: 1) reducing carbon emissions to the atmosphere, and 2) continued sequestration of carbon from the atmosphere over time. Our results revealed that the former pathway dominated the estimates over the relatively short accounting period, accounting for 97% of the total atmospheric carbon reduction (Table 3). A longer accounting period could likely increase the relative contribution of ongoing sequestration.

**Table 3:** **Carbon benefits (Gg, 1 Gg=10**^**9 **^**g) corresponding to area changes in Table**1

	**C Benefits**^**1**^	**Unprotected scenario (Gg) **^**2**^	
	**From**	**Less C**	**Addi**
**State**^**3**^	**Protection (Gg)**	**Emitted**	**C Fixed**
Alabama^+^	143	5,197	152
Arizona*	1,937	193	4
Arkansas^+^	2,606	3,343	116
California*	2,636	2,446	54
Colorado*	7,169	2,595	29
Connecticut	62	317	7
Delaware^#^	NA	113	3
Florida^+^	70	2,097	66
Georgia^+^	611	6,984	194
Idaho*	1,470	1,104	25
Illinois	516	620	7
Indiana	193	191	2
Iowa	0	248	4
Kansas^$^	NA	NA	NA
Kentucky^+^	251	2,546	69
Louisiana^+^	756	2,669	69
Maine	288	2,505	67
Maryland	97	485	10
Massachusetts	211	700	9
Michigan^$^	NA	NA	NA
Minnesota^$^	NA	NA	NA
Mississippi^+^	72	2,878	80
Missouri	748	2,239	33
Montana*	5,284	1,242	33
Nebraska^#^	NA	60	2
Nevada*	5,179	214	5
New Hampshire	295	342	5
New Jersey	361	194	5
New Mexico*	386	153	3
New York	1,771	1,954	36
North Carolina^+^	1,017	4,580	130
North Dakota^$^	NA	NA	NA
Ohio	120	1,037	29
Oklahoma^+^	369	1,461	47
Oregon*	8,120	7,911	513
Pennsylvania	227	1,324	33
Rhode Island	20	92	2
South Carolina^+^	231	3,465	92
South Dakota^#^	NA	60	1
Tennessee^+^	950	3,634	95
Texas^+^	570	5,944	168
Utah*	1,300	424	8
Vermont	84	219	3
Virginia^+^	1,477	2,899	78
Washington*	11,148	4345	243
West Virginia	166	960	23
Wisconsin	258	650	16
Wyoming*	7430	746	10
US48	66,599	83,380	2,580

^1^ State-level carbon benefits were estimated for forests in the protected areas compared to what could be emitted if there were no protection based on forest area lost rates in the corresponding state’s forests outside of protected areas. All carbon units are in gigagrams (Gg).

^2^ The predicted reduction in emissions of carbon (C), and additional C fixed if current net forest area loss rates were reduced by 20% in the 48 states’ unprotected forests.

^3^ See Table 1 for net deforestation rates in protected and unprotected forests in each state.

^+^ 13 Southern states (see Figure 2).

^*^ 11 Western states. The remaining states were in the Northern region.

^#^ States where forest area loss rates in the protected forests were larger than those in the unprotected forests. Therefore, calculation of presumed additional area loss in the protected forests using forest area loss rates in the corresponding unprotected forests (i.e., within the same state) were not applicable (NA).

^$^ States gained forest areas during the period, thus, reduction in forest area loss is not applicable (NA).

We had compared area change patterns in both afforestation and gross forest area loss categories by protection status. However, we based carbon benefits in our scenario analyses (e.g., 20% reduction of net loss rate between 1992 and 2001 calculated as (Area_aff_ – Area_def_) / Area_1992_ * 100), on net forest loss, that is, gross forest loss minus afforestation. If both the gross forest area loss rate and afforestation rate were used separately in the calculations of the carbon benefits analyses, it could theoretically improve the accuracy of estimates. However, there was no clear rationale for choosing which of the infinite number of combinations of changes in afforestation rate and gross forest loss rate that could be used to satisfy a fixed 20% reduction in net loss rate.

Protection of forestlands does appear to play a positive role in reducing carbon accumulation in the atmosphere. We found that up to 67 Tg C in the conterminous US could have been added to the atmosphere from the protected forests during the 9-year period had they not been protected (in other words, if forest area loss rate in unprotected forestlands was applied), at an annual rate of 7.4 Tg C. If net forest area loss rate in the 48 states’ unprotected forests was reduced by 20% (Table 3), over the 9 years, about 86 Tg C more could be stored in unprotected forestland (that is, lower carbon emissions plus additional fixation on forest remaining forest) for the study period, at an annual rate of 10 Tg C.

At the regional level, the South and West had a much higher potential to mitigate GHG emission than that of the North region by reducing forest area loss rate in unprotected forests (Table 2). For example, the South region alone accounted for 58% (49 Tg C) of the mitigated GHG emission in the conterminous US for the study period. This occurred by reducing forest area loss rate due to both the relatively high baseline area loss rate and large size in unprotected forestland. The potential of mitigating carbon emission to atmosphere in the West (22 Tg C) was 60% greater than that in the North (14 Tg C) although the size of unprotected forests in the West was 68% smaller than that in the North (Table 2).

By state, the top three in terms of the size of carbon benefits due to protected areas were Washington (11 Tg C), Oregon (8 Tg C), and Wyoming (7 Tg C) for the study period. The top three in terms of mitigating carbon emission by reducing net forest loss rate on unprotected areas were Oregon (8 Tg C), Georgia (7 Tg C), and Texas (6 Tg C). All these states are either in the South or West (Table 3).

### Relationships between population growth/density and forest area change rates

**Figure 3:**
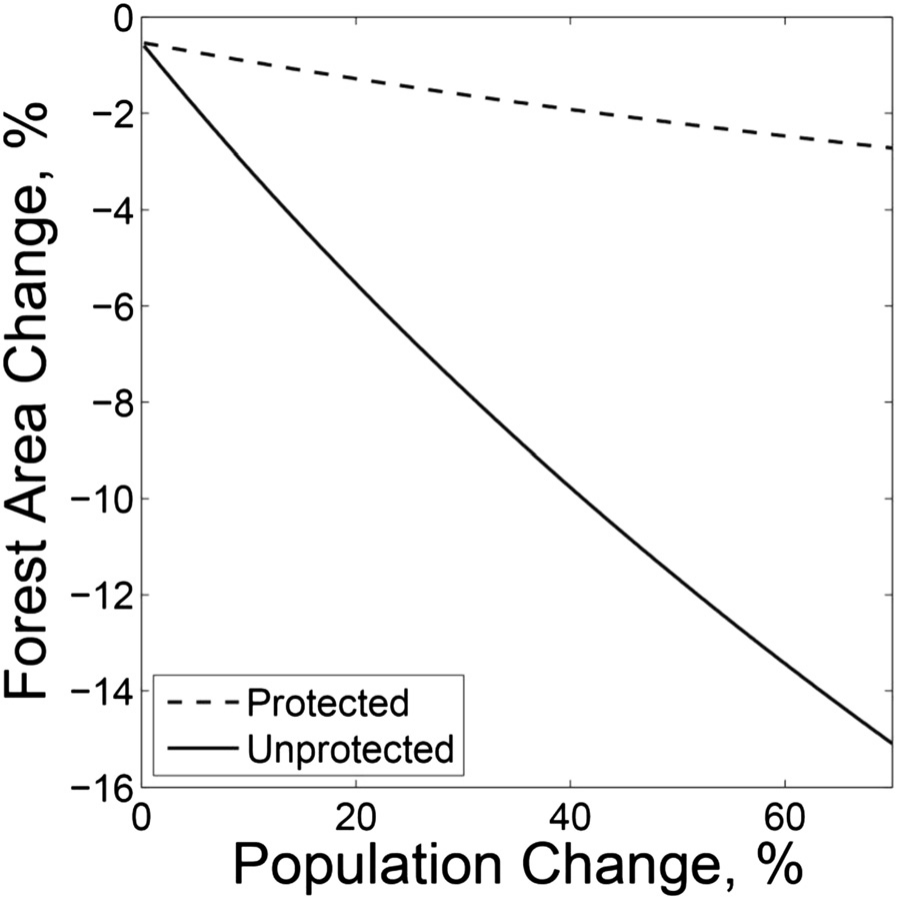
**General relationships between net forest loss rate ((Area**_**aff **_**– Area**_**def**_**) / Area**_**1992 **_**x 100) for the period of 1992 to 2001 and population change rate ((Population**_**2000 **_**/ Population**_**1990 **_– 1) * 100) by protection status at the state level, in terms of percent. An integrated model is shown in equation 1.

In the lower 48 states, overall population increased by 13.2% from 249 million in 1990 to 281 million in 2000 ranging at the state level from 0.5% in North Dakota to 66.3% in Nevada [22]. Mean US population density in 2000 was 36 inhabitants per square kilometer, ranging from 2 in the state of Wyoming to 438 in New Jersey. At the state level across the conterminous US, population growth had greater predictive power on forest area change rate than did population density. A general positive trend was detected -- that is, the forest area loss rate increased where higher population growth rates were observed. The greatest predictive relationship between forest cover change, population density and population change, and protection status included only population change and its interaction with protection status as fixed effects. Algebraically, the fixed-effects portion of the model can be written as

(1) $$y=-0.5269+\left(-27.4629+23.3201d\right)\left(\ln x_{2000}–\ln x_{1990}\right)$$

where *y* is the forest area change rate in percent, *d* is a dummy variable indicating protection status (0 for unprotected lands, 1 for protected lands) and *x*_*t*_ is population density in year *t*. This relationship is depicted graphically in Figure 3. As Figure 3 shows, the impact of population increases is much greater in unprotected than in protected forests. Although model selection was information-theoretic, we note that all effects retained in the model were also highly significant from a frequentist perspective (p<0.0001) and none of the effects excluded were statistically significant (p<0.05) in either the full model or subsequent reduced models. The fixed effects explained 62.1% of the variance in forest area change rate, while the random effect associated with state explained an additional 10.8%, with the true residual accounting for the remaining 27.2% of the variance. Although the land cover change rates are doubtless influenced by classification errors in both 1992 and 2001 NLCD maps from which the change map was derived, the strong statistical significance of the results, and the intuitive direction of the effects of protection status and population change contribute to the strength of the results.

**Figure 4:**
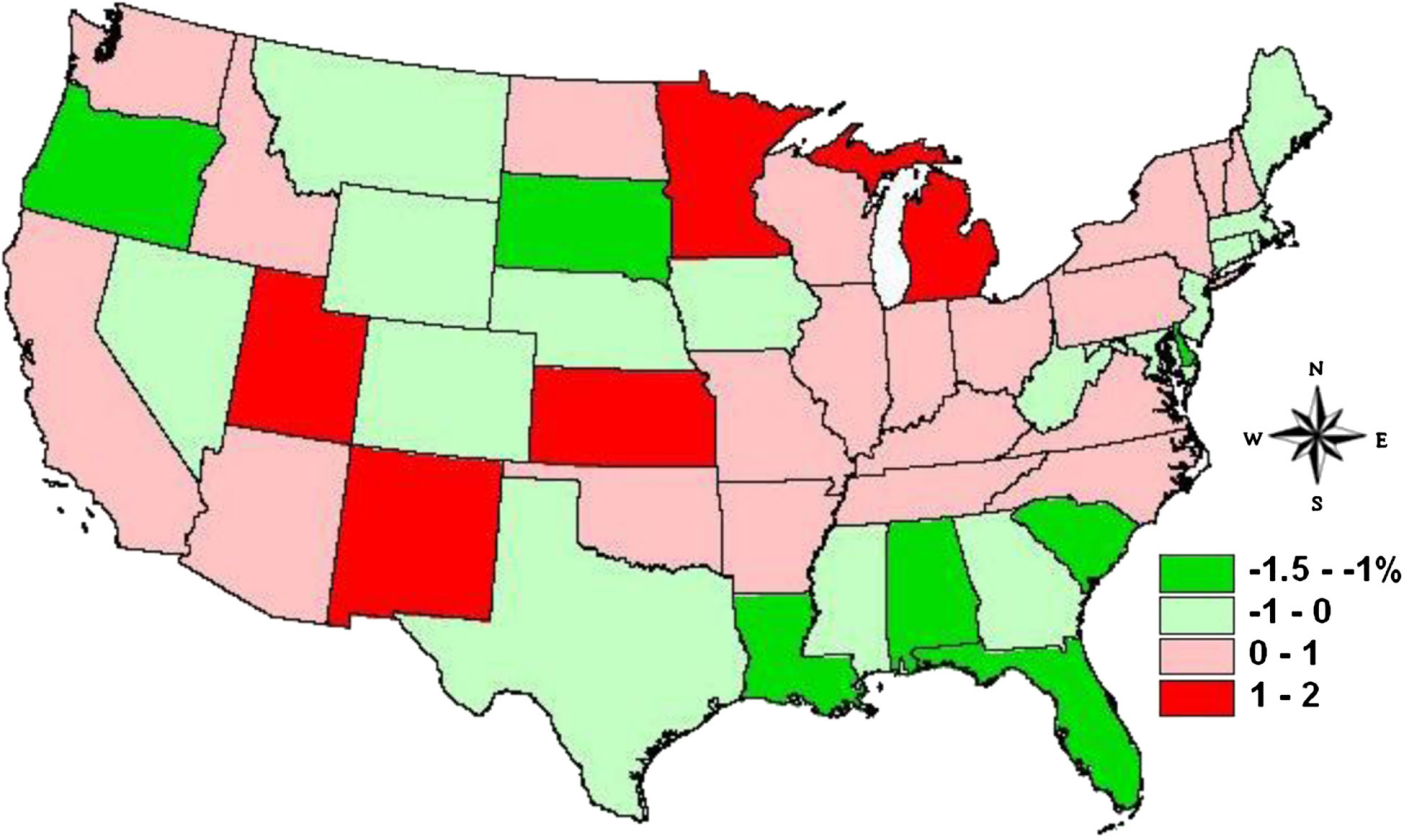
**State-level random effects from the mixed-effects regression analysis, which predicts net forest loss rates as a function of population change rates and protection status.** Greater negative values indicate higher rates of forest area loss, after accounting for demography and protection.

Spatial distribution of the random effects from the regression model at the state level is plotted in Figure 4. Although there is considerable noise in the data, the results suggest that rates of forest loss were higher in both protected and unprotected forests (i.e. change rate of forest cover was more strongly negative) across many of the coastal states in the southeastern US than would be expected from their population change. At the same time, interior states in the northern US experienced less forest loss than would have been expected given their demographic characteristics. Results in the Great Plains and in the western states showed no clear spatial pattern. Because forest loss indicated by NLCD will likely include disturbed areas that are actually forestland remaining forestland from a land use rather than a land cover perspective, the higher rates in the southeast likely suggest that some areas classified as forest conversion are actually disturbed areas that have temporarily lost forest cover but are not permanent conversion. This idea is supported by the larger rates of afforestation in unprotected forestlands of these states as indicated in Figure 2b.

## Discussion

This study provides an estimate of how much forestland would have been converted to nonforest in currently protected areas of forest, approximately 870 km^2^ for the study period in the conterminous US. Additional errors could be introduced simply because the protected areas can be established for a variety of reasons or goals and are not randomly assigned across the landscape [23,24]. In terms of carbon benefits, the resulting carbon benefit estimate is approximately 7 Tg C/yr, equivalent to the benefits estimated for a 10 year, $110 million per year tree planting program in the US [25].

Forest carbon benefits estimated from this study were based on 20% reduction scenario, which can serve as baseline information to infer other estimates for different reduction scenarios by assuming the relationship between reduction in net forest area loss rate and carbon benefits is linear and positively correlated (i.e., the higher reduction rate the more carbon benefits in general). Our estimated carbon benefits are also probably more realistic, consistent, and practical because our estimation is based on recent protected area data, forestland cover change rates, as well as projected population change in near future, at the national level.

Protection status in general matches well with ownership, but the correlation is not identical and varies spatially across the conterminous US. At the national level 95.5% of protected forestland was publicly owned whereas 97.7% of unprotected forestland was privately owned. This could be due to the fact that easements on private lands are generally not included in the Protected Area Dataset. However, these easements may not have biodiversity conservation as their main management objective. Furthermore, the percentage of ownership mixture varied by region. In the western US, 5.5% of unprotected forests were publicly owned and 1.7% of protected forests were privately owned. The patterns were reversed in the greater Eastern US, where 1.8% of unprotected forests were publicly owned while 8.5% of protected forests were privately owned. These uncertainties would inevitably affect calculations in forest area change rate and its variation associated with protection status because various owners likely implement different strategies in managing their forestlands [26-28]. For example, the average percentage of privately owned forestland in protected forests for the states of Delaware, Nebraska, and South Dakota, where forest area loss rates in protected forestland were higher than those in unprotected forests, was 9.8%, 119% higher than that of the national average (4.5%). However, its impact on the national carbon analyses is limited because all these states had a relatively small amount of forestlands. And more importantly, most of the nation’s carbon mitigation potential by reducing forest area loss rate comes from unprotected forestland that had a relatively lower mixture rate of public ownership (2.0%), compared to 4.5% mixture rate of private ownership in protected forestland. The overall effect of ownership mix on accuracy of estimating forest loss rate by protection status is likely limited due to small magnitudes of mixed percentage at the national level (i.e., about 2.0% ownership mixture in unprotected forestland and 4.5% ownership mixture in protected forestland, respectively).

This study revealed that our estimated carbon mitigation potentials due to forestland protection in unprotected forestland varied substantially by region. Several major factors could contribute to this variation: 1) afforestation and gross forest loss rate in unprotected forestland, 2) size of unprotected forestland, 3) forest carbon density, and 4) forest growth rate. For example, the size of unprotected forestland in West region was 68% smaller than that of North region, but its carbon mitigation potential was estimated 60% greater than that of North region (22 Tg C vs. 14 Tg C, Table 2). This was caused by 1) a much lower baseline forest area loss rate in general for the reduction in the North (−1.76%), compared to −6.64% in the West on average; and 2) a much higher forest carbon density and growth rate in the Pacific Northwest states and northern California [29,30] where unprotected forestlands were concentrated (Figure 1). In other words, even though the net forest area loss rates in the two regions were the same, the impact on carbon mitigation of reducing forest loss rate would be disproportionately increased in the region which has a higher forest carbon density and growth rate.

Analyses at the state level are coarse, but general relationships by protection status between net forest loss rate and population change rate (rather than population density) at state level were observed (Figure 3). There could be a number of reasons why an individual state might depart from the general trend. For example, Nebraska and South Dakota had only 5% and 8% of their relatively small forest area in protected status, leading to considerable uncertainty in the associated estimates for those states. Some states with high population density may even have little forestland available for forest area loss to begin with. For instance, the state of New Jersey had the highest population density in the 48 states (about 440 people per square kilometer) but showed relatively low forest area loss rate. This could be attributed to the facts that: a) its population is distributed unevenly (concentrated in the State’s northern areas), and significant forestlands occur in areas of low population density in the southern areas, and b) thirty-eight percent of forestland in New Jersey is publicly owned, the highest percentage of forestland in public ownership of any state east of the Mississippi [31]. This results in less forest available for forest loss [32] because in general the public land requirements limit forest conversion similar to protected area status. However these are not labeled protected areas because the lands are not protected for the purposes of biological diversity, as well as other natural, recreation, and cultural uses. Private land holders are subject to fewer restrictions.

At the national level, using the same carbon density for forestlands with different status (protected vs. unprotected) across a given state tended to underestimate the carbon benefits in the protected forestlands due to current protection by 23.9%, whereas it tended to overestimate the carbon benefits in the unprotected forestlands (assuming current net forest loss rate was reduced by 20%) by 4.5%. However, the overall effects on carbon benefit estimation are somewhat reduced by the uneven distribution of area proportions between protected and unprotected forestlands. For example, only about 12.1% of the FIA plots used in sensitivity analysis were located within the protected areas (the remaining plots were located within unprotected areas). Possible error effects of using the same carbon density for benefit estimation were relatively higher in the eleven western states than that for the remaining states because a higher proportion of forestlands, on average, was protected in the western states.

Although we used net forest loss rate in this study to be able to estimate carbon benefits associated with protection status and a reduction scenario, we also examined general patterns in afforestation and gross deforestation rates between protected and unprotected forestlands. This improved our understanding for the study period at the state level that protection not only had a reduced gross forest loss rate, but also had a reduced afforestation rate.

Net forest area loss rate in the 48 states’ unprotected forests in general was much greater than that in the protected forests, with very few exceptions. The states of Delaware, Nebraska, and South Dakota were the three states where net forest area loss rates in the unprotected forests were smaller than those in the corresponding protected forests (Figure 2a). However, their impact on national-level calculations was limited due to their relatively small forestland areas. The potential to reduce forest area loss rates in the 48 states’ unprotected forestland varies by region, with the greatest potential being in the Southern region due to a combination of high loss rates, rapid growth, and a large fraction of forests in the unprotected category.

Strategies for reducing forest loss rates, and thereby providing atmospheric carbon mitigation, based on policy options addressing unprotected forestland should reflect the magnitude and sources of land use change. For example, annual forest change rates in unprotected forestland during the 1992–2001 study period was −0.45%, equivalent to about 5,800 km^2^ loss per year. By comparison, the projected mean forest loss to urban development alone up to 2050 is about 1800 km^2^ per year across the conterminous US [33], or approximately 30% of current forest loss in unprotected forests. In other words, the maximum reduction scenario from its current loss in the 48 states’ unprotected forests could not possibly exceed 70% without significant changes in the magnitude and pattern of urban development due to population increases. Moreover, urban development is not the only factor driving land use conversion in forested lands. As a result, a 70% reduction from current forest loss rate in the 48 states’ unprotected forestland is probably unrealistic. If we assume a maximum reduction scenario of 50% of the current forest loss rate within the 1.3 million km^2^ unprotected forests, that would result in 2,900 km^2^ forestland conversion per year. While well beyond the current basic need for urban development, such a scenario probably reflects an optimistic upper bound. Under that scenario, atmospheric carbon content could be reduced by 24 Tg C/yr, equivalent to 1.5% of the nation’s total carbon emissions in 2007 from fossil fuel combustion including commercial, industrial, residential, transportation, and electric power [34].

## Conclusions

Our study provided primary quantification of carbon benefits from current protected areas for biodiversity conservation and other uses for forests across the conterminous United States, an amount equivalent to equivalent to the 7 Tg C/yr carbon benefits estimated for a 10 year, $110 million per year tree planting program in the US. Protected areas are not only associated with lower forest area loss rates, but also with lower afforestation rates in general at the state level. Results from our study suggested that the potential integrated carbon benefits, based on maintaining current protection status and future implementation of a 20% reduction in forest area loss in unprotected forestlands, would be equal to 9.4% of current forest ecosystem net carbon sequestration in the conterminous United States. Our estimates can be linearly extrapolated to other scenarios based on the methodology applied in our analyses, assuming no leakage effects or interacting market effects from reduced harvest levels. Forest area change dynamics are negatively correlated with population dynamics at the state level in general and tend to be more evident in forestlands that are not in protected areas. More research is needed to estimate carbon benefits more precisely. Detection of forest area change obtained from land cover maps covering a longer period may be preferred for certain analyses. There are many interactions and tradeoffs in the system between variables such as forest area, land use change, carbon per area, disturbance rates, management actions, and harvested wood products, and a more detailed analysis would be needed to capture these effects.

## Methods

This study covers the 48 states in the conterminous US. Regional divisions (Figure 2c) were mainly based on similar histories of forestland use [35] and for consistency with another study focusing on forest carbon changes at the same scale [17]. We used two primary data sources which are described further below: 1) remote sensing based NLCD Retrofit Change Map [14] to calculate net forest area change, and 2) Protected Area Dataset of the United States (PADUS, version 1.1) released in June, 2010 [36] to distinguish protected forestlands from unprotected forestlands. We used a third dataset of forest carbon density and growth tables [29] based on USDA Forest Service, Forest Inventory and Analysis (FIA) program data to convert forest area change to carbon change. A fourth dataset, population data at the state level, was obtained from US Census Bureau [22]. The dataset was used for obtaining population density data in both years of 1990 and 2000 (persons/km^2^), and estimating population change rates for the period. We used the same population change rate for both protected and unprotected forestlands for each of the states due to limitation of the Census data (e.g., non-spatial statistics).

### National land-cover change map

Forest conversion based on cover change was determined using the 30-m NLCD 1992–2001 Retrofit Change Map, generated by the Multi-Resolution Land Characteristics Consortium [14]. The product was developed to provide more accurate land cover change data than would be possible by direct comparison of NLCD 1992 and NLCD 2001, using a hybrid change analysis process incorporating both post-classification and specialized ratio differencing change analysis techniques. The change map identifies eight primary classes: 1) open water, 2) urban, 3) barren, 4) forest, 5) grass/shrub (G/S), 6) agriculture, 7) wetland, and 8) ice/snow at Anderson Level I [37] at the beginning year and for the ending year. For example, class 46 indicates the land was changed from forest in 1992 to agriculture in 2001. We excluded ice/snow related classes in this study, as their area is trivial (0.02% of the total conterminous US, all within the western states). Overall classification accuracy in the NLCD 1992 map was 80.4% while it was improved to 85.3% in the NLCD 2001 map [38].

Forest areas (including deciduous, evergreen, and mixed) in 1992 and 2001 for each of the 48 states were determined using the NLCD Retrofit change map (1992–2001). We obtained three forestland status at the ending year of 2001 from the change map: 1) afforestation, 2) deforestation, and 3) forest remaining forest. Thus, forest areas in 1992 could be calculated as deforestation plus forest remaining forest whereas the forest areas in 2001 were summed from afforestation and forest remaining forest. Forest area change for each state was determined using the NLCD Retrofit.

It is noted that the forest area changes calculated from different data sources such as remote sensing and field plot data inventory based datasets (e.g. FIA) may differ, and each has distinct advantages and limitations. Differences in methodology and definitions between the approaches are nontrivial, and the results must be interpreted in context of the approach. Land cover refers to the physical and biological cover over the surface of land that may be observed by remote sensing and can lead to reasonable questions about the precision of detected changes in forest cover or whether loss of forest cover is forest conversion or temporary loss of cover due to harvest. Land use includes social and economic perspectives within which lands are managed [18] and the forested areas with only a temporary loss of cover due to harvest or other disturbance will continue to be classified as forest although forested areas near urban development may be designated as nonforest. However, remote sensing based observations tend to be more efficient in identifying forest area change across large scales over time [14,17,39]. Over the period of interest, the national forest inventory design changed, so for this analysis, we chose to use the remote sensing based dataset for area change because it was a consistent dataset over the time period. Forest area changes in different protection status were calculated based on the protection status from the Protected Area Database described below.

### Protected area database

The PADUS 1.1 is a national geo-database that is an inventory of protected areas mostly owned by public agencies or non-profits, and defined as being “Dedicated to the preservation of biological diversity and to other natural, recreation and cultural uses, managed for these purposes through legal or other effective means.” [36]. PADUS1.1 does not include lands protected from conversion under conservation easements, which means that estimated forest loss reduction in lands designated as protected is likely an underestimate. However, protection for biological diversity is but one of a number of reasons for the use of conservation easements, so not all easements would be included in this type of analysis. The lands in PADUS 1.1 are assigned conservation status codes that both denote the level of biodiversity preservation for each protected area, and indicate other natural, recreational and cultural uses. One of the PADUS 1.1 missions is required to organize and assess the management status (i.e. apply GAP Status Codes) of elements of protecting areas for biodiversity.

There are four general GAP classes in the PADUS 1.1 ranging from the most land protection (class 1) for biological diversity and other uses to the least land protection (class 4). The 4 classes are [36]: 1) an area having permanent protection from conversion of natural land cover and a mandated management plan in operation to maintain a natural state within which disturbance events (of natural type, frequency, intensity, and legacy) are allowed to proceed without interference or are mimicked through management; 2) an area having permanent protection from conversion of natural land cover and a mandated management plan in operation to maintain a primarily natural state, but which may receive uses or management practices that degrade the quality of existing natural communities, including suppression of natural disturbance; 3) an area having permanent protection from conversion of natural land cover for the majority of the area, but subject to extractive uses of either a broad, low-intensity type (e.g., logging or recreation) or localized intense type (e.g., mining). It also confers protection to federally-listed endangered and threatened species throughout the area; and 4) there are no known public or private institutional mandates or legally recognized easements or deed restrictions held by the managing entity to prevent conversion of natural habitat types to anthropogenic habitat types. Any other forestlands that were not classified explicitly in the dataset were considered unprotected forests in this study (Personal Communication, Lisa Duarte, USGS GAP Analysis Program, June 2010). Our carbon benefits related analyses were focused on the forestlands in two extreme status (protected and unprotected) that contained 71% of the total conterminous forest area detected from the NLCD Change Map.

Preliminary analysis suggested that net forest area change rates between the GAP classes 1 and 2 from the PADUS1.1 did not significantly differ. Thus, we reclassified these two classes into a category we call protected lands. We described class 3 as partially protected (forestlands fall in this category was not included in the carbon analyses), and class 4 as less protected. The forest change rates between the less protected class 4 category and unprotected forestland were not significantly different. The less protected category was only 2.2% of the forested area of the US (Figure 1), and the area of unprotected forest was about 61% of forestland in the conterminous US. Thus, we used forest change rates in unprotected forestland (excluding less protected lands) for all related calculations throughout the analyses because using the unprotected category for comparison minimizes local potential effects on change-rate estimates.

### Data analyses

Previous studies have demonstrated that calculating afforestation and deforestation separately can refine overall carbon estimates [12,40]. This study, however, used net forest area change rate as a baseline reference to simplify estimation of effects in reducing forest loss on carbon mitigation. We derived areas of afforestation, forestland becoming nonforest, and forestlands remaining forestlands from the change map within each protection status at the state level, then calculated the net area change rates in terms of percent between 1992 and 2001 as (Area_aff_ – Area_def_) / Area_1992_ * 100 based on forest area in 1992. Our analyses, however, were focused on changes of net forest area loss between protected and unprotected forest categories. State-level analyses were aggregated to the national level as needed. We converted area change to carbon change under assumed management scenario (20% reduction of net forest loss rate) using carbon density data described below.

To estimate how much more forest would have been cleared in the protected areas if the lands were not protected, we applied the forest area loss rates detected in the corresponding unprotected areas within the same state. To illustrate spatial patterns of differences in forest area change rate between protection status, we calculated the difference as Rate_protected_ – Rate_unprotected_ for each state. We linked the determined population variables with forest area change rates (focusing on the net forest area change but also looking at gross forest area change for general comparison purposes).

From one perspective, no hypothesis testing is needed in this study to interpret the results because the input data exhaustively cover the study area. As a result, there is no sampling uncertainty associated with the estimated forest area change rates. However, the actual NLCD data reflects not only the mechanisms driving forest area change but also data limitations, such as classification errors. Moreover, the actual change can be viewed as one change outcome taken from a set of alternative outcomes that could have occurred. States are large ensembles of 30-m pixels, sufficient in size to average a great many sources of positive spatial autocorrelation, such as differences in economic and policy drivers of development and forest area change between local regions and metropolitan areas. For an overall test, we used Friedman’s nonparametric test [41], with forest area change rate as the dependent variable, protection status as the independent variable, and state as a block. Pairwise comparison between different protection status was conducted using the Wilcoxon-Nemenyi-McDonald-Thompson post-hoc test as described by Hollander and Wolfe [42]. All analysis were performed using R [43]; post-hoc tests employed code by Galili [44].

Carbon sequestration by that area of forest remaining forest due to 20% reduction in net forest loss assumption was calculated using forest carbon density and growth data identified at the state level based on FIA data by various common-group forest types [29]. We used the same carbon density data across each state and performed sensitivity analysis using available field plots from the FIA to evaluate the potential effect of such application on our carbon benefit estimation by protection status. To calculate carbon loss from forest becoming nonforest, we used a conversion factor of 0.8. This factor was based on the assumption that 80 percent of the nonsoil forest C (including live tree, stand dead, understory, down dead wood, and forest floor) would be removed from the ecosystems and eventually lost to the atmosphere during conversion to nonforest. Carbon sequestration for forest remaining forest, or for forests that avoided conversion under rate reduction scenarios, was estimated using forest growth rates determined by effective mean forest ages for each state. Effective mean forest ages at the state level were inferred from mean live tree carbon density data for a given state [29].

To better understand the relationship between human population density and growth, rates of change in forest cover, and protection status, we used a linear mixed-effects modeling approach to predict the rate of change in forest cover between 1992 and 2001. We initially estimated a full model using log-transformed population density in 2000, change in log-transformed population density between 1990 and 2000, protection status, and the interactions of these three variables as fixed effects, and state as a random effect (because we have two values for each state, one for protected areas and one for unprotected areas). Then, we employed backward selection with the Akaike Information Criterion (AIC) to find the best-fitting model [45]. Residual plots and qq plots were used to check against heteroscedasticity and non-normality of the residuals. The random effects associated with state-level departures were exported and mapped for visualization and examination.

We also estimated how much more forest would have remained forest, and how much carbon emissions could have been reduced, if land use policies designed to reduce forest area loss (either by restrictions, or through incentives) were implemented in the 48 states’ unprotected forests. We used a basic scenario approach similar to Gullison et al. [7], who applied two reduction scenarios (by 20% and 50%, respectively) in their study of tropical forest removals in relationship to climate policy. For the percent reduction in our study, we considered that a complete cessation of forest loss is not a realistic option simply due to projected demand for land conversion caused by population growth [44]. The US population is projected to have an increase of 42%, from 310 million in 2010 to 439 million in 2050 [46]. Alig et al. [33] estimated that across the conterminous US about 1,800 km^2^ forestlands would be needed for urban development alone per year up to 2050. It is reasonable to assume that much of this land conversion would occur in unprotected forestlands.

The NLCD data suggest that average rate of forest conversion to nonforest in the unprotected forestlands between 1992 and 2001 was about 5,800 km^2^ per year across the conterminous US. A reduction of 20% results in 4,640 km^2^ such a conversion per year, which is larger than the estimated conversion demand for urban development alone (1,800 km^2^) because other associated demands in forestland conversion resulting from population growth also exist. Thus, we based our analysis on a 20% reduction in net forest loss within the 48 states’ unprotected forestlands to provide reasonable room for meeting other demands in forestland conversion associated with future population growth.

However, the effects of reduction in net forest loss on forest area and carbon dynamics are linear, assuming other factors, such as forest type and growth rate, are constant. Therefore, effects of reductions at levels other than 20% can be obtained by multiplying the impacts by an appropriate factor. For example, the effects of 40% reduction on changes in forest area and carbon could be estimated by multiplying the effects of this 20% reduction by 2.

## Competing interests

We do not have competing interests.

## Authors’ contributions

DZ contributed to the overall study design, data analyses, and wrote the manuscript. LH contributed to manuscript development including the initial idea for the study, constructive discussions and writing. MD contributed to the study methods, quantitative analyses, and writing. All authors read and approved the final manuscript.

## Authors’ information

DZ is Research Scientist II in Department of Natural Resources & the Environment, University of New Hampshire, USA. LH is Research Forester in USDA Forest Service, Northern Research Station, Durham, New Hampshire USA. MD is Professor in Department of Natural Resources & the Environment, University of New Hampshire, USA.
